# “Can you recommend a journal for my paper?”

**DOI:** 10.1007/s40037-022-00712-0

**Published:** 2022-05-02

**Authors:** Olle ten Cate

**Affiliations:** Utrecht Center for Research and Development of Health Professions Education, Utrecht University Medical Center, Utrecht University, Utrecht, The Netherlands

“Good question! Let’s have a dialogue. I just received an invitation from* Perspectives on Medical Education* to write a commentary on Rees et al.’s article about journal choice [1]. Our lunch chat is a great basis for this commentary. So okay, authors want to be read, of course. While quantity of readers alone may be less important than the *right* audience, you still want many readers in your audience of interest. Accordingly, my question for you is: which audience should read your work?”

## “Medical educators in general. But maybe first: what is the best medium for my message?”

“Until not so long ago writing a book was the preferred channel for science, after millennia of oral knowledge transfer. For centuries, book owners were owners of knowledge, and libraries temples of knowledge, the preferred whereabouts for scholars. Then journals, with regular appearance and subscribers, became more convenient: new knowledge was simply delivered to your door mat. Now the internet is quickly replacing books and printed journals. Many excellent health profession education (HPE) books still appear, and I belong to those buying them, but I have mixed feelings about their impact. Even high-quality chapters, usually written by invitation, are read by too small an audience. Were they published as journal articles, they would probably be better read and cited. I have written books, but seldom meet people who read, let alone own them. A book is not a sensible option for your manuscript.”

## “But journals evolve too, right?”

“Well, the future of publishing may look quite different from now [2]. Major medical journals already include films, animations, graphical abstracts, audio interviews and podcasts. But journals are still the prime channel of scientific information. Yet, subscriptions have become less relevant because access is increasingly open to anyone. *Perspectives on Medical Education *pioneered this in HPE, by offering diamond open access, i.e., without costs for readers or authors. Yet to find articles, you now search for authors or topics, rather than journals. I use Google Scholar a lot, more than good-old PubMed. Topics and authors come first, and I look at the journal of a paper to gauge its credibility. But browsing email alerts of quality journals does make me aware of interesting things I was not looking for. Alerts are my preferred subscription.”

## “You mentioned one journal, but how many publish HPE articles?”

“Unlike decades ago, the choice of an HPE journal is rather an *embarras de choix *than being limited in number [3]. I keep track and have a list of at least 30 options, plus another 90 if you look more widely and internationally. Feel free to download it (https://tinyurl.com/53zh3ddu) [4]. Rees et al. found that 4000 randomly selected HPE articles across 2019 and 2020 were published in 233 journals, but included specialty journals that only occasionally publish education articles [1]. Clearly these lists will not help you directly. In an attempt to delineate the HPE playing field, Maggio et al. identified the 24 most credible journals for medical education (the ‘MEJ-24’) [5]; mostly general, but some with specific focus (anatomy, surgery, simulation). This is an excellent first cut if medical education is your domain of interest.”

## “But twenty four is still a lot. Please be more specific.”

“Think of making a list of three, and anticipate rejections, as the top HPE journals accept 15% or less of all submissions. Rees et al. found that successful authors value things like the journal’s audience, editor reputation, impact, access, speed of processing, and the fit of their manuscript with the journal’s scope [1]. For fit, look at the journal’s aims and scope and the types of articles it features, and browse a few recent issues. If you still hesitate, email the editor directly. I recently did that, with a preliminary abstract, and received a helpful answer the same day. Also look where important references in your piece were published. Open access helps to reach a wider audience, but that usually costs an article processing charge (APC) after acceptance, for HPE journals up to $3500.”

## “You did not mention the journal impact factor (JIF). Shouldn’t that be criterion #1?”

“Ha! You touch on a critical issue here. The JIF does reflect whether the journal’s articles are being cited, but don’t let that determine your decision. Metrics also have limitations. Any metric of quality that becomes a target in itself ceases to be a good measure and will be gamed (Campbell’s and Goodhart’s Laws) [6]. If a researcher’s prime goal is to appear in high impact journals, the true purpose of research gets lost, so don’t stare blindly at JIFs. But shooting for a top journal is good if your study meets three conditions: high rigor in methods, unique findings and excellent writing style.”

## “Journals are much slower than other media. My friend’s paper took a year before it appeared. Should I look for journals with fast procedures?”

“Wait. Remember that quality takes time. Scientific journals can be trustworthy only if they scrutinize the quality of articles. My grandfather, born in 1892, cherished the credo ‘if printed, it must be true’. Now, in times of uncertainty and ‘fake news’, credibility must be earned, and serious scientific literature has the moral obligation to maintain that standard. Peer review is not perfect, but it is the best method we have and it takes time. In quality journals, articles have been critically reviewed by at least three to five scholars. Journals that offer rapid publication should be distrusted. For example, today I received four personalized invitations from such journals to submit a manuscript, adding to another 13 in the previous four days, all from different journals. Such predatory journals promise fast reviews, high impact and rapid open access publication, but are really just after your APC. Do not respond, even if personalized and referring to one of your previous articles. Serious processing takes time. Expect to wait at least six months before seeing a successful paper published online, prior to issue assignment. What is new is the option to deposit unreviewed empirical work as a preprint on an open access repository, such as arXiv.org, bioRxiv.org or ResearchSquare.com. These serve the rapid exchange of scientific advances, and some HPE editors encourage this [7, 8].”

## “Do review processes and editorial processes differ?”

“Yes! Even among credible journals. If you have never published, you would not know it, but if you have, you can see that editorial decisions may just refer to review comments, may include personal comments of an associate editor, and may, on top of that, include comments of the Editor-in-Chief who ultimately makes the decision. The ‘lazier’ journals have the reviewers do all the work by just confirming their recommendations, and having them review the revisions too, without forming an original editorial opinion—not much editing in their editor roles! I prefer journals with quality editorial processes. In some cases, these journals help editing, explain how they arrive at decisions and value reviews by publishing their criteria [9–13]. Take a look at these.”

## “I hope to get really good feedback. So which journal selects the best reviewers?”

“Basically, you can’t tell. Reviewers may be good or not so good, and editors should not always follow their advice; they should replace reviewers who make superficial, brief or derogatory comments. Reviewer quality is not something I think of when choosing a journal. As a regular reviewer myself, my judgments are not different for different journals, but sometimes I comment ‘this seems better for a different type of journal’. Having said that, top journals likely attract better reviewers. When pressed for time I tend to accept review invitations from the most credible journals, leaving others, sadly, with more difficulty finding reviewers. Some journals encourage reviewers to engage junior researchers. I must say, writing my reviews with others improves their quality. So why don’t you join me in an upcoming review?”

## “Fine, but for my own paper, don’t you even have a biased opinion about the best HPE journal?”

“Ah, you are challenging me now. The answer is no, but I can’t help but have a biased sympathy for *Perspectives*, the journal that originated in the Netherlands!”
